# Editorial: Vesicular transport, the actin cytoskeleton and their involvement in virulence mechanisms during host-parasite interaction

**DOI:** 10.3389/fcimb.2023.1229067

**Published:** 2023-06-14

**Authors:** Rosario Javier-Reyna, Yunuen Avalos-Padilla, Sabrina Marion

**Affiliations:** ^1^ Department of Infectomics and Molecular Pathogenesis, Center for Research and Advanced Studies-National Polytechnic Institute (CINVESTAV-IPN), Mexico City, Mexico; ^2^ Nanomalaria Joint Unit, Barcelona Institute for Global Health (ISGlobal, Hospital Clínic-University of Barcelona), Barcelona, Spain; ^3^ Nanomalaria Group, Institute for Bioengineering of Catalonia (IBEC), The Barcelona Institute of Science and Technology, Barcelona, Spain; ^4^ University Lille, CNRS, Inserm, CHU Lille, Institut Pasteur de Lille, U1019-UMR 9017-Center for Infection and Immunity of Lille, Lille, France

**Keywords:** host-parasite interactions, actin cytoskeleton, vesicular transport, virulence factors, extracellular vesicles

## Introduction

Host-Pathogen interactions involve finely regulated mechanisms contributing to the establishment of infection (Méthot and Alizon, 2014). Upon contact, signaling cascades are induced in both the microorganism and the host with different objectives: i) the invading microorganism aims to enter the host, establish itselfand cause damage, ii) the host aims to prevent pathogen’s entry and persistence. Thus, both protagonists expose their attack and defense strategies. In the aforementioned events, participation of the actin cytoskeleton and the endomembrane system is essential in both the host and the pathogen.

Multiple microorganisms manipulate the host cytoskeleton by remodeling actin filaments and microtubules to facilitate invasion or to escape host defenses (Morrissette and Sibley, 2002; Wen et al., 2020; Dutta et al., 2022). The actin cytoskeleton of parasites also participates in host cell entry (invasion), cell migration (motility), transport of molecules between organelles (vesicular transport), and phagocytosis (acquisition of nutrients), all these cellular processes being crucial for the virulence of the infectious agent (Kumar et al., 2014; Mansuri et al., 2014; Manich et al., 2018; Bañuelos et al., 2022).

In line, bacteria have developed attack nanomachineries, such as injectosomes and molecular nanosyringes. These structures are of protein nature and allow toxins or bacterial effectors to be injected into target cells, which facilitates entry or induces host cell destruction (Ruano-Gallego et al., 2015; Wagner et al., 2018).

Eukaryotic cells have a system of interconnected endomembranes that is highly conserved between species (Gurkan et al., 2007; Grissom et al., 2020). This system is involved in protein degradation, recycling and secretion, notably through the formation of extracellular vesicles (EVs). The content of EVs is diverse, and include RNAi, antigenic and major histocompatibility complex molecules, among other cargos. EVs play an important role in short or long distance communication between cells and tissues, and contribute to diverse biological processes such as regulation of cell viability and modulation of immune responses (Abels & Breakefield, 2016; Chidester et al., 2020; EL Andaloussi et al., 2013; Munhoz da Rocha et al., 2020; Engin, 2021).

Advances in microscopy techniques, biochemical, cellular and molecular biology, proteomics, bioinformatics, and other fields have enabled the generation of new knowledge to better understand the molecular strategies employed by microorganisms and the host in their ongoing battle for supremacy. This Research Topic presents recent findings describing how different parasites: *Entamoeba histolytica*, *Echinococcus granulosus*, and *Bastocystis* as well as the bacteria *Salmonella enterica* modulate host responses by various processes such as actin rearrangement and secretion of EVs to promote host cell entry or tissue invasion.

## First contact and the actin cytoskeleton

When microorganisms infect host cells, they induce a complex response to promote their survival and counteract the host defense mechanisms. In most cases, this process is associated morphological changes in the host cell. For instance, *S. enterica* infection of enterocytes results in actin cytoskeleton remodelling leading to microvilli effacement. The study led by Felipe-López et al. demonstrated that this process involves the translocation of effector proteins via the *Salmonella* Pathogenicity Island 1-encoded type III secretion system (SPI1-T3SS). They employed live cell imaging to study the dynamics of host-bacteria interactions, and identified two distinct mechanisms of microvilli effacement. The first, F-actin depolymerization mediated by villin, and the second, cytoplasmic G-actin consumption by formation of membrane ruffles. Moreover, this study also shows that the SopE effector protein is a key factor in the destruction of the intestinal barrier during *Salmonella* infection.

Pathogenic microorganisms have also evolved strategies to sequester host molecules and use them for their own benefit. As an example, Pacheco-Sánchez et al. described *Entamoeba histolytica*’s interaction with host acetylcholine through a 150 kDa intermediate subunit of the Gal/GalNAc lectin. They showed that this interaction triggers the activation of parasite’s GTPases leading to rearrangement of the cytoskeleton and enhanced vesicular trafficking, which contributed to parasite proliferation, ultimately promoting host tissue invasion (Pacheco-Sánchez et al.).

## Extracellular vesicles, a key component of host-pathogen interactions

In recent years, EVs released from microorganisms or from infected cells have been well recognized as mediators of intercellular communication and immune modulators through EVs incorporation by recipient cells. Using proteomic approaches, Shi et al. characterized host-derived EVs released in the plasma of infected mice at different stages of infection by the parasite *E. granulosus*. They found distinct EV compositions at 7 and 20 weeks post-infection, corresponding to the conversion from protoscoleces to persistent hydatid cysts. Interestingly, EV proteomes included important *E. granulosus*-related proteins and immune regulatory effectors. They also investigated the effect of plasma isolated EVs on splenic mononuclear cells, and observed that EV are internalized by B and T cells and display immunomodulatory functions. Co-cultural experiments showed that 7 weeks-EVs upregulated the relative abundance of regulatory T cells and increased the expression of IL-10.

In a different study, Leonardi et al. investigated EVs released from virulent strains of the protozoan parasite *Blastocystis.* They reported that *Blastocystis* subtypes 7B and 7H secrete EVs with physical characteristics and protein components similar to other microorganisms, and showed that these EVs modulate *in vitro* host cell responses and impact on the growth of gut bacteria species. Notably, *Blastocystis* ST7 EVs induce death in HT29 cells and modulate THP1 cell inflammatory cytokines. Thus, these studies add to our understanding of the importance of EVs in regulating host responses to persistent parasite infections.

## Conclusions

The vast and constantly expanding knowledge on the functions of the actin cytoskeleton, receptor/ligand molecules, endomembrane communication and transport system, as well as extracellular vesicles in both the pathogen and host, has opened up new possibilities for the development of diagnostic, therapeutic and preventive approaches. Modern tools such as genomics, proteomics and metabolomics have allowed the identification of new target molecules for vaccine development, which can be exploited to develop novel strategies to combat pathogens that pose a threat to public health.

## Author contributions

All authors listed have made a substantial, direct, and intellectual contribution to the work and approved it for publication.
